# A Multiple Relevance Feedback Strategy with Positive and Negative Models

**DOI:** 10.1371/journal.pone.0104707

**Published:** 2014-08-19

**Authors:** Yunlong Ma, Hongfei Lin

**Affiliations:** Information Retrieval Laboratory, School of Computer Science and Technology, Dalian University of Technology, Dalian, Liaoning, China; Xiamen University, China

## Abstract

A commonly used strategy to improve search accuracy is through feedback techniques. Most existing work on feedback relies on positive information, and has been extensively studied in information retrieval. However, when a query topic is difficult and the results from the first-pass retrieval are very poor, it is impossible to extract enough useful terms from a few positive documents. Therefore, the positive feedback strategy is incapable to improve retrieval in this situation. Contrarily, there is a relatively large number of negative documents in the top of the result list, and it has been confirmed that negative feedback strategy is an important and useful way for adapting this scenario by several recent studies.

In this paper, we consider a scenario when the search results are so poor that there are at most three relevant documents in the top twenty documents. Then, we conduct a novel study of multiple strategies for relevance feedback using both positive and negative examples from the first-pass retrieval to improve retrieval accuracy for such difficult queries. Experimental results on these TREC collections show that the proposed language model based multiple model feedback method which is generally more effective than both the baseline method and the methods using only positive or negative model.

## Introduction

Since the inherent limitations of current retrieval models, it is nearly impossible for any retrieval model to return satisfactory results for every query. Indeed, a query might be so simple or ambiguous that a large number of top-ranked documents are non-relevant, and we usually call it difficult query. In such a case, a user would have to either reformulate the query or go far down on the ranked list to examine more documents. Both may decrease the user satisfaction. As a result, improving the effectiveness of search results for such difficult queries would bring user satisfaction which is the ultimate goal of search engines.

The language modeling approach to text retrieval was first introduced by Ponte and Croft in [1] and later explored in [2]–[4]. The relative simplicity and effectiveness of the language modeling approach, together with the fact that it leverages statistical methods that have been developed in speech recognition and other areas, make it an attractive framework in which to develop new text retrieval method. Although the language modeling approach has performed well empirically, a significant amount of performance increase is often due to feedback [1], [2], [5]. When a user is unable to submit an effective query (which happens often in informational queries due to [1], [2], [5] insufficient knowledge about the relevant documents), feedback can be quite beneficial with the basic idea of extracting useful terms or features from relevant (or pseudo relevant) documents and use them to expand the original query or update the query model. The feedback techniques can help not only text retrieval but also multimedia retrieval [6], such as image searching [7], landmark searching [8] and etc. Although several kinds of feedback techniques, including relevance feedback [9]–[11], pseudo-relevance feedback [12]–[14] and implicit feedback [15], have been extensively studied in information retrieval, most existing work on feedback relies on positive information, i.e., exploiting relevant documents or documents that are assumed to be relevant. To our knowledge, both of implicit feedback and pseudo-relevance feedback have limitations individually. An explicit feedback operation is harmful to user's experience, and the hypothesis of pseudo relevance feedback, the top 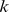 documents in the first-round retrieval are all relevant to a specific query, is often invalid [16] which can result in a negative impact on the retrieval performance.

In this paper, we focus on a real environment that a user submits one query to a search engine and then clicks several hyperlinks of return list for viewing. Fortunately, the click operations can be recorded with the form of search engine query logs. Thereby, we assume that all the clicked documents are all highly relevant and others in the return list before the lowest-ranked clicked document are irrelevant. Then, according this assumption, we use the positive information in the relevant document to derive a new positive model that expands the original query. We choose the Relevance-Based Language Models (RM) in [17], which is a typical language model based Query Expansion (QE) approach in [18], as the implementation of positive model estimating approach in our work.

Some previous studies concluded that when positive documents are available, they are generally more useful than negative documents in [19], so the positive feedback has been studied extensively. As a result, how to exploit negative documents for feedback has been largely under-addressed, and negative feedback has just attracted attention recently. In [16], [20], the authors studied different methods for negative feedback using only irrelevant information and neglecting all relevant information. Intuitively, if we can learn from both of positive and negative information to raise the rank of relevant documents and prune non-relevant documents from the original ranked list concurrently, we will improve the performance more. In this paper, we tackle this challenge and estimate a negative feedback model by considering not only the negative information but also the positive model which we just obtained using an improved RM approach. Finally, we use the multiple relevance feedback strategy which is formed by the fusion of positive and negative relevance model to rerank the unseen list. To evaluate the effectiveness of the proposed method, we construct a test collection containing only appropriate queries from TREC collections. Experiment results show that the proposed multiple relevance feedback strategy is effective for improving ranking accuracy and it outperforms the one using only either positive or negative feedback.

The rest of the paper is organized as follows. In the next section, we review related work firstly. Section 3 describes our feedback framework for language models. Then, in the section 4, we show our positive and negative model estimating approaches in details. Section 5 contains experimental results, as well as a discussion of those results and the last section is a conclusion.

## Related Work

### Relevance Feedback

Relevance feedback has been shown to be effective with different kinds of retrieval models in [14], [15], [21], [22]. In the vector space model, feedback is usually done by using the Rocchio algorithm, which forms a new query vector by maximizing its similarity to relevant documents and minimizing its similarity to non-relevant documents [10]. The feedback method in classical probabilistic models is to select expanded terms primarily based on Robertson/Sparck-Jones weight [9]. Unfortunately, both of them cannot be naturally implemented in the language modeling approaches [16]. In the language modeling approaches, relevance feedback can be implemented through estimating a query language model [22] or relevance model [17] through exploiting a set of feedback documents.

Recently, several query expansion techniques have been developed in the language modeling framework, including, e.g., mixture-model feedback method [22] and relevance model [17]. The basic idea is to use feedback documents to estimate a better query language model. Both the mixture model and relevance model have been shown to be very effective, but the relevance model appears to be more robust [23]. In the mixture-model feedback, the words in feedback documents are assumed to be drawn from two models: (1) background model and (2) topic model. The mixture-model feedback finds the topic model that best describes the feedback documents by separating the topic model from the background model. The topic model is then interpolated with the original query model to form the expanded query. Much like mixture-model feedback, the relevance model also estimates an improved query language model. Given a query 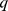, a relevance model is a multinomial distribution 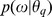 that encodes the likelihood of each term 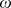 in the query as evidence. To estimate the relevance model, the authors first compute the joint probability of observing a word together with the query words in each feedback document and then aggregate the evidence by summing over all the documents. It essentially uses the query likelihood 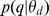 as the weight for a document 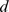 and takes an average of the probability of word 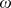 given by each document language model.

When there are no real relevance judgments available, alternatively, pseudo relevance feedback [12]–[14] may be performed, which simply assumes that a small number of top-ranked documents in the initial retrieval results are relevant and then applies relevance (positive) feedback. Thus, both of the two above feedback approach in the language model are based on this assumption and our work differs from those in that we use real relevance judgments instead of the assumption above.

There are lots of pervious work focus on explicit feedback which can be used to obtain user's judgements leading to a good performance retrieval, but unfortunately, quite few users will put up with an additional interactive operation. Even if we also use the user's judgements in this work, but a main difference of our work from the explicit feedback approach is that the additional operation is dispensable and we just use the information extracted from search engine query logs. This idea is similar to the measures proposed in [15] which is named implicit feedback, but our work considers not only positive information but also negative information, and this research field has just attracted attention recently.

### Negative Feedback

There have been some attempts to exploit non-relevant documents. Query zone [24] appears to be the only major heuristic proposed to effectively exploit non-relevant information for a document routing tasks. It shows that using non-relevant documents that are close to the original query is more effective than using all non-relevant documents in the collection. Also, the work in [25] exploits high-scoring documents outside of top 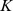 documents (called pseudo-irrelevant documents) to improve the performance of pseudo-relevance feedback. The work in [16] and later extension [20] exploit the top non-relevant documents to improve the ranking of documents and they are the earliest studies of negative relevance feedback in the language modeling framework. The last one defines an important concept called generalization of a language model and the authors propose an optimization framework based on this concept. It is a brilliant work and we propose our feedback strategy in this paper also based on the same concept, but we consider that positive feedback model should be taken into account when optimizing the negative model to more aggressively (but carefully) prune non-relevant documents, leading to a more effective multiple relevance feedback method.

## Multiple Feedback Framework for Language Model

### Problem Formulation

Given a query 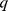 and a document collection 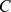, a retrieval system returns a ranked list of documents 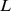 where 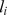 is the 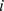-th ranked document in the ranked list 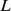. We assume that the query is difficult enough so that there are only a handful of relevant documents 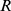 in top 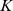 ranked documents (seen so far by the user) 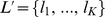 and most of documents in 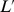 are non-relevant. The goal of our study is to use these positive examples 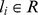 to build a positive language model 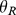 first which describe the information need more accurately, so that the rest unseen relevant documents will be assign a higher relevance score when reranking.

However, the second part of our feedback model is a set of negative models. Therefore, we use all the negative feedback examples, i.e., 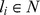 to build a set of negative language models, each corresponds to a negative example. Then, every negative language model will be optimized by taking account of the original query model and the positive language model, so that these improved negative language models are better able to describe other unseen non-relevant documents and improve the ranking of relevant documents by pushing down non-relevant documents in the ranked list.

More formally, given a specific query 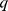, a ranked list 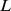 and a set of relevance judgements including relevant (positive) documents 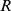 and non-relevant (negative) documents 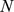 corresponding this query 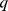, our goal is to estimate a positive language model 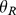 (then combine with the original query language model 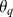 to form a relevance topic language model 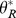) and a set of improved negative language models 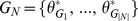, where 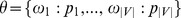, i.e., each language model consists of words along with their probabilities. All the models above can then be plugged into the final feedback strategy to improve feedback performance.

### The KL-Divergence Function

In this paper, we only focus on the positive and negative feedback problem in the language modeling framework, so we just use Language Model (LM) as the basic retrieval model in all our work. There are two main score functions in LM, the original and basic one is Query-Likelihood (QL) function [26]. In it, we construct from each document 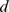 in the collection a language model 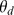. The goal is to rank documents by 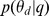, where the probability of a document is interpreted as the likelihood that it is relevant to the query. Using Bayes rule we have:
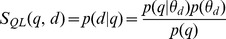
(1)where 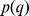 is the same for all documents, and so can be ignored. The prior probability of a document 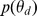 is often treated as uniform across all 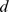 and so it can also be ignored. Thereby, return results ranked by simply 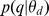, the probability of the query 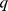 under the language model derived from 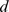. The Language Modeling approach thus attempts to model the query generation process: documents are ranked by the probability that a query would be observed as a random sample from the respective document model.

The other score function named KL-divergence function [27] which is one of the most effective score function in the language modeling framework [23]. It is a generalization of the query-likelihood function and would score a document 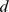 w.r.t query 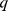 based on the negative Kullback-Leibler divergence between the query language model 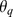 and the document language model 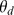:
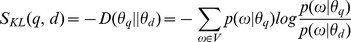
(2)where 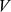 is the words in the vocabulary.

Clearly, the two main tasks are to estimate the query language model 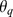 and the document language model 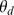. The document language model 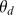 is usually smoothed using Dirichlet prior smoothing which is an effective smoothing method [28].

The query model intuitively captures what the user is interested in, thus would affect retrieval accuracy significantly. The query language model 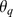, is often estimated (in case of no feedback) based on:
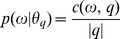
(3)where 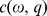 is the count of word 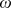 in query 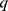 and 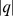 is the total number of words in the query. Such a model, is not very discriminative because a query is typically extremely short. When there is feedback information, the information would be used to improve the estimate of query language model 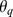.

According to all pervious work, all our work use language model with KL-divergence score function as the basic retrieval model throughout this paper.

### The Positive Feedback Model

#### The Relevance-Based Language Model (RM)

In order to describe users' information need more effectively, we have to estimate a positive feedback language model first, and the Relevance-Based Language Models (RM), which is a typical pseudo relevance feedback (PRF) approach implementation in the language modeling framework, is chosen as the basic of the positive model estimating approach in our work.

In RM estimate function, except the PRF document models, the document weight consists of two components: a document relevance score and a document prior. The former represents the initial document relevance probability, while the latter is the prior probability of selecting the corresponding document. More formally, for each given query 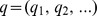, based on the corresponding PRF document set 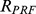, the RM estimates an expanded query model:
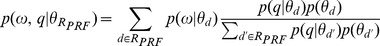
(4)where 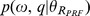 is the estimated relevance model. A number of terms with top probabilities in 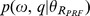 will be used to estimate the QE model (i.e. the expanded query model).

In Equation 4, 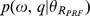 is the probability of a term 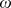 in the language model 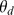 for a document 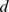, 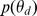 is 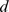
's prior probability, and 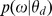 is the query-likelihood:
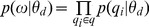
(5)


In RM, the weighting function is:
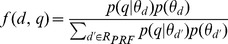
(6)where the QL relevance score 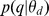 and document prior 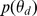 are integrated to form the document weight. The 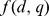 plays a key role in RM since it distinguishes the RM from a mixture of document language model (see 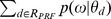).

To apply revised weighting functions under the RM framework, we re-formulate the RM as:
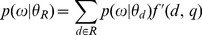
(7)where 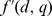 denotes any revised document-weighting function that satisfies 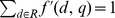, and different 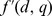 will derive different RM implement.

#### Adaptation of KL-Divergence as the Document Weight

According to Equation 6, 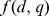 in RM is a normalized query-likelihood score (see Equation 1) being eliminated the constant 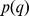 and since the document prior 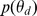 is assumed to be uniform, it turns out that the weighting function is the normalized query-likelihood probability:
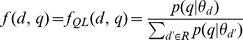
(8)


The normalized query-likelihood document weight 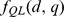 are called as QL weights in the following text. From Equation 2 and 8, the QL weights can out be computed efficiently, because it lead to a additional calculation operation. Moreover, the KL-divergence is a more effective function in information retrieval tasks. Thus, we adapt the KL-divergence function as the document weight in the original RM function:
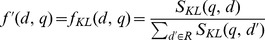
(9)


When the first time ranked list return, all the necessary KL scores 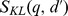 can be obtained at once, and then, we can figure out the normalized KL weight very soon.

Nevertheless, RM is a typical pseudo relevance feedback (PRF) approach and the basic assumption is the a small number of top-ranked documents in the initial retrieval results are relevant. So, it is reasonable to assign each document weight by their relevance score descending sequence. But in our work, all the relevant document are extract from truly judgements by user's feedback, so we consider that document which got a lower relevance score in the first time retrieval maybe need more attention and higher weight in feedback processing, because it is necessary to improve the new query description ability for the document which have not be described well by the original query. Thus, we modify the KL document weight as follow:
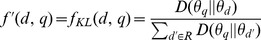
(10)and the final RM function we use as the positive feedback model is:
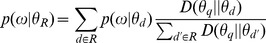
(11)


The experiment results in the evaluation section show that the our positive feedback model (named RM-KL) is more effective than RM.

### The Negative Feedback Model

The basic idea in relevance feedback is to extract useful information from positive documents and use them to update the original query language model as we have done above. When a query is difficult, it is often impossible to obtain a lot of (or enough) positive documents for feedback. Therefore, the best way would be to exploit the negative documents to perform negative feedback [16]. The idea of negative feedback is to identify distracting non-relevant documents and penalize unseen documents containing such irrelevant information.

The two negative feedback methods proposed in [20] are SingleNeg and MultiNeg methods which we briefly describe below.

#### SingleNeg

This method adjusts the original relevance score of a document with a single negative model. Let 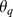 and 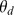 be estimated query model and document model, respectively. Let 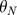 be a negative language model estimated based on negative feedback documents 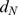. The new scoring according to this model is:
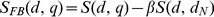
(12)


In order to estimate 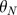, it is assumed that all non-relevant documents are generated from a mixture model of a unigram language model 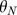 and a background language model (generating common words). The log-likelihood of the 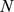 sample documents is:
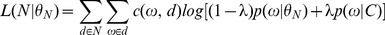
(13)where 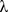 is a mixture parameter that controls the weight of the background model. A standard EM algorithm is used to estimate parameters 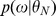.

#### MultiNeg

This method adjusts the original relevance score with multiple negative topic models. Document 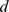 w.r.t query 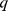 is scored as follows:
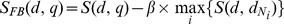
(14)where 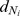 is a negative document representation and 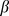 is a parameter that controls the influence of negative feedback. EM algorithm is used to estimate a negative model 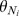 for each individual negative document 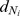 in 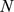. Then 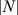 negative models be obtained and combined with the above formula for reranking.

According to the experimental results and conclusion in [20], the MultiNeg strategy lead a better performance than the other one, so of course, we choose the MultiNeg as our basic negative feedback modeling strategy. Specifically, based on the KL-divergence score function, the MultiNeg formula will be expended to the following form:
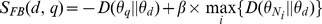
(15)and we use it in our experiments.

## Feedback Language Model Optimization

### The Goal of Optimization

**Figure 1:**
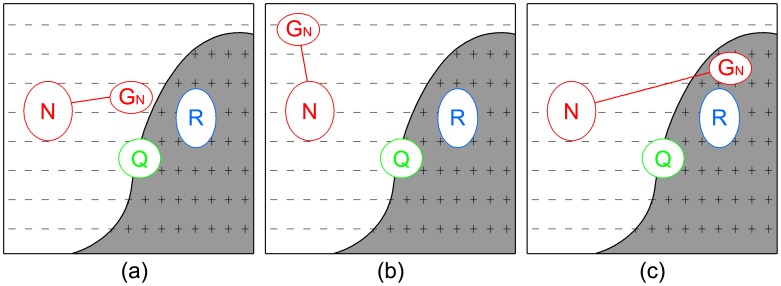
Examples of General negative language model.

A main goal of our study is to improve the estimate of the positive and negative document language models. A effective positive language model can combine with the original query language model to improve the ranking of relevant documents by boosting their relevance scores directly, and it can be optimized through the EM algorithm. A effective negative document language model can be used to exploit the top non-relevant documents to improve the ranking of documents, and it can be obtained by generalizing a basic negative document language model with an optimization framework. There are three criteria have to be considered in the optimization process: (1) closeness to the original negative language model (to ensure the accuracy), (2) closeness to the relevance (positive) language model (if it is far from the information need, the pruning power is not very effective), and (3) a generalization constraint. The reason why all these three components are important can be explained in Figure 1, where (a) shows that the general negative language model is safe and effective since it is both close to the original negative language model (thus ensures that the pruned documents to be non-relevant) and reasonably close to the relevance language model (thus can make a difference in the top-ranked results through pruning).

In the next section, we present an optimization framework for improving the estimate of both positive and negative document language models.

### The Framework of Optimization

In order to build a more general negative language model, we need an optimization framework that given 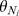, searches in the space of all language models and finds a set of more general negative language models, i.e., 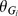, finally, picks out the best model, i.e. 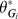.

Therefore, we prefer the objective function definition and expend it with positive feedback model which is a important pair in our work as follows:
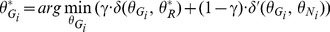
(16)where 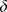 and 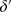 are divergence functions. 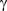 is a tradeoff between closeness to the relevant topic model and closeness to the original negative model.

We also continue to use the restriction to avoid over-generalization:
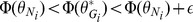
(17)


It provides that general negative language model can deviate 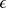 at most from original negative language model. The generality 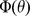 is defined as:
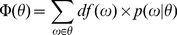
(18)where 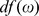 is the number of documents containing word 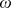 in collection 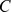 (document frequency) and 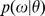 is the probability of word 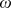 given language model 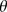.

Next, we describe the divergence functions, 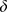 and 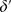 in the optimization framework.

### Divergence Functions

We define both of the two divergence 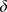 and 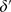 in Equation 16 based on KL-divergence. First, the divergence from general negative model to the relevant topic model is KL value exactly:
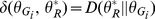
(19)


The KL-divergence function also be called as relative entropy, the former variable in 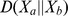 is consider as the truly distribution and later variable is testing distribution. But, it is unreasonable to consider either 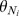 or 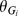 as the truly distribution, so we continue to use the symmetric version of KL-divergence [27] for the divergence between general negative model and the original negative model.
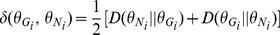
(20)


With these instantiations, the objective function is completely defined.

### Shrinkage of Searching Space

In the objective function (Equation 16), the searching space is infinite, and in order to find an optimal solution efficiently, we make it tractable by searching in a finite space of all feasible solutions, 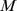. Therefore, we propose two steps for shrink the searching space, and we describe them in details here.

#### Conflict Removing

As we have explained in Section 4.1, the goal of general negative language model optimization is 1) close to the original negative language model 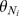 (the first part of Equation 16), 2) and close to relevance topic language model 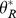 (the second part). The closeness to 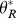 ensures the pruning power, but the original negative language model is in collision with the relevance topic model (the same terms with high observation frequency), that is the main reason that these negative documents are returned in the top-rank list. So we remove the terms, which have a high probability in relevance topic model 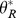, from the original negative language model 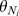. Specifically, top 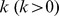 terms in 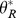 be removed in our experiments.

#### Term Elimination

Similar to the Perturbation step in [29], foreach 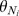 in the original negative language model set, we build a more general negative language model 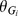 by removing appropriate terms 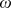. But in our work, we remove those terms iteratively that satisfy 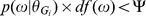, with the increment 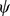 of 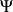 for iteration, until minimizing the objective function and it is no doubt that the revise negative language model is still more general than 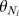. Table 1 shows the iteration of term elimination.

**Table 1:** Algorithm of Term Elimination for Optimization.

Algorithm 1. Term Elimination for Optimization.
**Input:** a set of negative language models 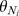
**Output**: a set of general negative language models 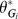
**Parameter**: the increment 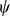 of 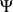
Initialize parameter 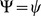 and 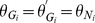
for 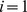 to 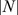 do
repeat
Set 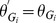
foreach 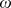 satisfies 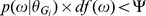
Remove 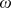 from 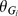
end foreach
Normalize the probabilities in current 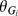
Update 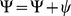
until the distance of 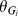 is higher than 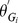 (Eq. 16)
Set 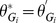
end for

Note that, after any term removing, the probabilities are re-normalized to ensure they are comparable.

## Evaluation

### Experimental Data Set

The evaluation is done using two standard TREC (Text REtrieval Conference – http://trec.nist.gov/) collections: Robust04 and GOV2, that are representative of heterogeneous and homogeneous data sets, respectively, with the details in Table 2.

**Table 2:** Data statistics of Robust04 and GOV2.

Corpus	Docs	Original Q	Final Q	First-pass P@20
Robust-04	528,155	No.301–450	112	0.163
Gov-2	25,205,179	No.701–850	129	0.229

Our first data set is Robust Track of TREC 2004 which has 528,155 news articles. We use 150 queries in this set for our experiments. The Robust Track is a standard ad hoc retrieval with an emphasis on the overall reliability of IR systems which contains difficult queries and is a heterogeneous data set. The data set is called “Robust04” in the following text.

The second data set is a TREC test collection for use in the Terabyte Track which is a homogeneous data set. It contains 25,205,179 documents crawled from the “.gov” domain sites in 2004, and there are 150 queries in this set. The data set is called “GOV2” below.

For both data sets, preprocessing of documents and queries involves only stemming with Porter stemmer and removing stopwords by a minimum English stopwords list in Lucene (Apache Lucene – http://lucene.apache.org/).

Since our goal is to positive and negative feedback in language modeling framework, we construct a simulated query set to simulate the users' behavior on a search engine. Because there is no truly feedback information, so in our experiments, we treat the relevance judgements published by TREC as the feedback by several truthful users. Considering the hypothesis of multiple relevance feedback, the relevance and non-relevance documents have to appear concurrently in the feedback judgement, so we filter both two query set above following the constraint is that: the baseline method (the language model with the KL-divergence score function and Dirichlet Prior Smoothing, more details in Section 5.2) returned at least 1 relevant document in top 20 (user clicked) and at least 1 non-relevant document (user swept over) before the lowest-ranked relevant document in top 20 also. Finally, there are 112 and 129 queries are available respectively for our experiments, with more details in Table 2. In particular, we treat all topic titles as queries and neglect their description field.

### Baseline

In order to evaluate the effectiveness of our method, we use three methods as the baselines for comparison.

1. The Language Model was implemented by the Indri (Indri Toolkit – http://www.lemurproject.org/indri.php) toolkit, in which the Dirichlet smoothing prior 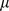 is set to 2000 for Robust04 and 1500 for GOV2 empirically [26], and this method is denoted by LM-Dir.
2. The Relevance-Based Language Model was also implemented by the Indri, which is one of the PRF expansion approaches and only use the positive feedback model, based on the query-likelihood method by [17] and denoted by RM-QL.
3. The MultiNeg feedback method which we implement following the describe in [20], considering only the negative feedback information, and we denote it by MultiNeg (details in Subsection 3.4). All the parameters are set to the empirical value (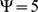, 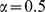 and 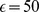) [29].

### Experiment Procedure

The multiple relevance feedback strategy which we proposed in this paper, take account of both positive and negative feedback information. Therefore, the goal of our experiments is to simulate a scenario when a user has viewed the top-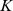 ranked documents (on the first page). He (or she) has clicked a few hyperlink for further view and is about to view the rest of the search results (click the button of next page). At this point, we can naturally apply feedback information to re-rank all the unseen documents. As we have showed in Section 5.1, we set 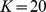, which simulates the scenario of applying feedback, the relevant and non-relevant documents have been found on the first page of search results and the user is about to view the next page of results.

In order to set parameters in our method, i.e., 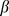 (described in Section 3.4, a parameter to control the influence of the negative feedback) and 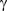 (described in Section 4.2, a tradeoff between two divergence functions), we do a 5-fold cross validation as follows: we fix the number of positive feedback terms 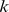 (has been described in Section 4.4) and 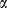 (described in [17], a parameter to control the influence of the positive feedback) to 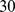 and 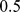 respectively, then learn both of two parameters based on the training data. The other parameters are set the same value to the Multi-Neg (described in Section 5.1).

When the user clicked the next-page button, the top-20 ranked documents have been browsed by the user, so they should not be returned again on the next pages. To simulate this scenario and reflect the performance directly, we remove the top-20 documents in the original ranked list from the reranking results.

Specifically, we denote the positive feedback strategy method (described in Section 3.3.2) by RM-KL, and the final multiple relevance feedback strategy method we proposed by Multi-FB.

## Results

**Figure 2:**
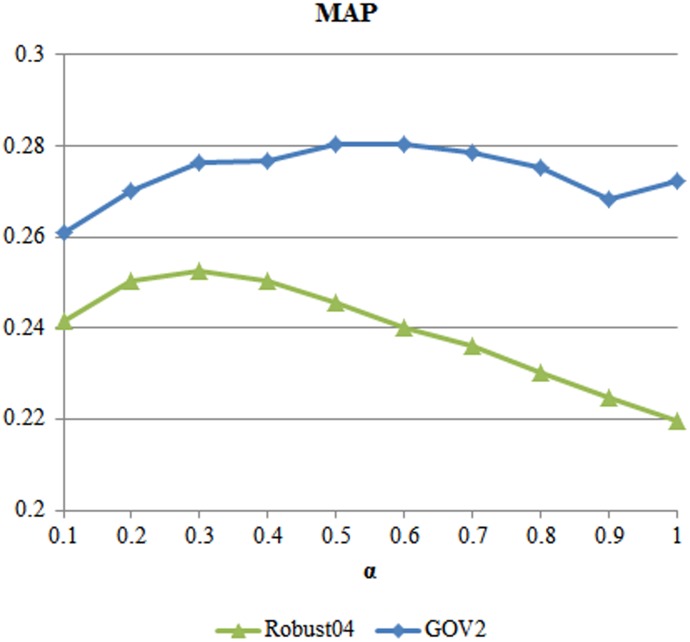
MAP performance of RM-KL for all collections when M = 30.

Before doing some detail testing, Figure 2 shows the results of assigning different 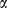 on two TREC collections, when 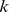 (the number of positive feedback terms) is set to 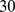 empirically. As it can be seen in Figure 2, the RM-KL method perform well when the value of 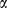 is set to 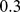 for Robust04 and 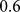 for GOV2. Thus, we set the parameter to optimal values above for our RM-KL and Multi-FB methods.

With the setup showed above, the top-ranked 1000 unseen documents for all runs were compared in terms of two sets of performance measures: Mean Average Precision (MAP) and Precision at 20 (P@20), which reflect the utility from users perspective who can not bear with more than two pages browsing. Please note that MAP is considered as the main measure, however, we show our experimental results based on all measures for the sake of completeness.

Finally, in order to see the effectiveness of our proposed strategies, we compare them with the three baselines methods after several ad-hoc retrieval testing on two TREC standard collections and list Table 3 and Table 4 to show the results with MAP and P@20 measures, respectively. Table 3 shows the cross validation results with MAP and Table 4 also show cross validation with P@20, based on both collections Robust04 and GOV2, respectively. These Tables also show the results of assigning different value to 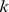 for every collections. The MAP of our method Multi-FB is 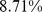 and 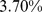 higher than the Relevance-based Model (RM-QL) based on pseudo relevance feedback, also 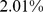 and 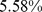 higher on P@20 for Robust04 and GOV2 respectively.

**Table 3:** MAP scores of various methods.

Meth.	MAP				
Robust-04					
LM-Dir	0.220	0.220	0.220	0.220	0.220
RM-QL	0.241	0.249	0.253	0.254	0.254
RM-KL	0.252	0.259	0.260	0.260	0.256
Multi-Neg	0.229	0.229	0.229	0.229	0.229
Multi-FB	**0.262**	**0.266**	**0.266**	**0.262**	**0.260**
Gov-2					
LM-Dir	0.272	0.272	0.272	0.272	0.272
RM-QL	0.270	0.277	0.280	0.281	0.281
RM-KL	0.277	0.281	0.282	0.283	0.282
Multi-Neg	0.276	0.276	0.276	0.276	0.276
Multi-FB	**0.280**	**0.282**	**0.286**	**0.285**	**0.285**
**k**	**10**	**20**	**30**	**40**	**50**

**Table 4:** P@20 scores of various methods.

Meth.	Precision@20				
Robust-04					
LM-Dir	0.354	0.354	0.354	0.354	0.354
RM-QL	0.347	0.348	0.354	0.356	0.356
RM-KL	0.347	0.349	0.351	0.357	0.356
Multi-Neg	**0.356**	**0.356**	0.356	0.356	0.356
Multi-FB	0.352	0.355	**0.357**	**0.361**	**0.360**
Gov-2					
LM-Dir	0.467	0.467	0.467	0.467	0.467
RM-QL	0.467	0.464	0.469	0.473	0.466
RM-KL	0.470	0.476	0.480	0.485	0.482
Multi-Neg	0.468	0.468	0.468	0.468	0.468
Multi-FB	**0.479**	**0.486**	**0.490**	**0.493**	**0.492**
**k**	**10**	**20**	**30**	**40**	**50**

According these results, we can see the Multi-FB method outperform the RM-QL and Multi-Neg in most case, it shows that taking account of positive and negative feedback information concurrently lead to a more effective feedback language model than using either of them singly. We also find out that the RM-KL method we proposed preforms better than the RM-QL method, it confirms the effectivity of KL-divergence in information retrieval field.

## Conclusion

Because of the inherent limitations of current retrieval models, it is nearly impossible for any retrieval model to return satisfactory results for every query. The feedback is an important and useful technique for tackling this challenge, which can be done automatically without requiring extra user effort based on implicit feedback information. In this paper, we focused on a scenario that a user submitted to a search engine and then clicked several hyperlinks of return list for viewing. Thus, we addressed the problem of data sparseness in feedback, with the assumption is that all the clicked documents are all highly relevant and the others in the return list before the lowest-ranked clicked document were irrelevant, by proposing a multiple relevance feedback strategy in the language modeling framework using KL-divergence score function. In the strategy we proposed, we learned a positive language model and a set of general negative language model from the feedback documents. Finally, we used the multiple relevance feedback strategy which is formed by the fusion of positive and negative language models to rerank the unseen list for users. In the last section, our experiments showed that the proposed multiple relevance feedback strategy is effective for improving ranking accuracy and it outperformed the methods using only either positive or negative feedback.

There are a few limitations of our work. First, all the experiments are based on simulated feedback instead of real world relevance feedback by truly users, so a future work will be to test the proposed methods with real feedback data. Second, maybe some other feedback method can be interpolate into our multiple strategy later. The last, because of the great number of combination possibility, we do not have enough time and equipment to do optimization for all parameters in our experiments. So we will pay more attention on all of these in our future work.
